# 6,6′-Dimeth­oxy-2,2′-[(cyclo­hexane-1,2-di­yl)bis­(nitrilo­methyl­idyne)]diphenol

**DOI:** 10.1107/S1600536810033465

**Published:** 2010-08-28

**Authors:** Qian Zhang, Peng-Fei Yan, Guang-Ming Li, Peng Chen

**Affiliations:** a School of Chemistry and Materials Science, Heilongjiang University, Harbin 150080, People’s Republic of China

## Abstract

The mol­ecule of the title compound, C_22_H_26_N_2_O_4_, has two azomethine linkages, both of which are in an *E* configuration. The cyclo­hexyl ring adopts a chair conformation. The dihedral angle between the benzene rings is 66.57 (9)°. The mol­ecular structure is stabilized by two intra­molecular O—H⋯N hydrogen bonds.

## Related literature

For related structures, see: Aslantaş *et al.* (2007 ▶); Tozzo *et al.* (2008 ▶).
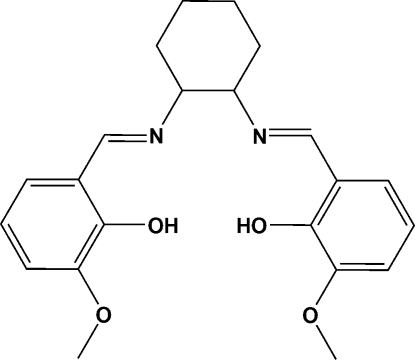


## Experimental

### 

#### Crystal data

C_22_H_26_N_2_O_4_
*M* *_r_* = 382.45
Monoclinic, 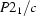

*a* = 15.014 (3) Å
*b* = 12.029 (2) Å
*c* = 12.099 (2) Å
β = 107.54 (3)°
*V* = 2083.5 (6) Å^3^
*Z* = 4
Mo *K*α radiation
μ = 0.08 mm^−1^
*T* = 293 K
0.22 × 0.20 × 0.15 mm

#### Data collection

Rigaku R-AXIS RAPID diffractometer
19987 measured reflections
4748 independent reflections
2352 reflections with *I* > 2σ(*I*)
*R*_int_ = 0.062

#### Refinement

*R*[*F* ^2^ > 2σ(*F* ^2^)] = 0.072
*wR*(*F* ^2^) = 0.164
*S* = 1.03
4748 reflections
259 parameters
1 restraint
H atoms treated by a mixture of independent and constrained refinement
Δρ_max_ = 0.13 e Å^−3^
Δρ_min_ = −0.18 e Å^−3^

### 

Data collection: *RAPID-AUTO* (Rigaku, 1998 ▶); cell refinement: *RAPID-AUTO*; data reduction: *CrystalClear* (Rigaku/MSC, 2002 ▶); program(s) used to solve structure: *SHELXS97* (Sheldrick, 2008 ▶); program(s) used to refine structure: *SHELXL97* (Sheldrick, 2008 ▶); molecular graphics: *SHELXTL* (Sheldrick, 2008 ▶); software used to prepare material for publication: *SHELXL97*.

## Supplementary Material

Crystal structure: contains datablocks global, I. DOI: 10.1107/S1600536810033465/ng5006sup1.cif

Structure factors: contains datablocks I. DOI: 10.1107/S1600536810033465/ng5006Isup2.hkl

Additional supplementary materials:  crystallographic information; 3D view; checkCIF report

## Figures and Tables

**Table 1:** Hydrogen-bond geometry (Å, °)

*D*—H⋯*A*	*D*—H	H⋯*A*	*D*⋯*A*	*D*—H⋯*A*
O3—H3⋯N1	0.88 (2)	1.77 (2)	2.572 (3)	150 (3)
O4—H4⋯N2	0.86 (3)	1.80 (3)	2.587 (3)	152 (3)

**Fig. 1.**
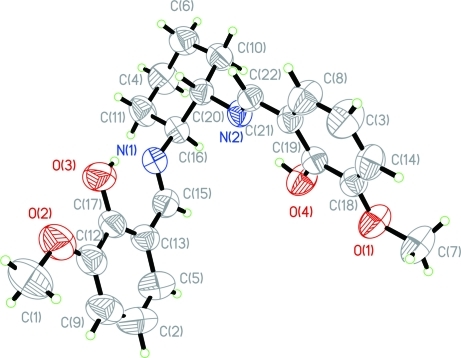
The molecular structure of the title compound, showing 50% probability displacement ellipsoids.

## supplementary crystallographic information

### Comment

We present the crystal structure of the title compound, as shown in Fig. 1. X-ray analysis suggests that the imino group is in the *trans* configuration and the two aromatic rings are lying on in front of the other. Bond lengths and angles within the aromatic rings are consistent with reported examples. Short H-bonds exists between the OH groups and the imino groups in *ortho* position.

### Experimental

The title compound was prepared by a known method. *o*-Vanillin (2 mmol, 0.304 g) in acetontrile (20 ml) and *trans*-1,2-cyclohexanediamine (1 mmol, 0.114 g) in methanol (20 ml) were mixed and refluxed for about 4 h at 358 K. The reaction mixture was cooled and filtered; Compound was obtained by crystallization from a mixture methanol/acetonitrile solution after a few days. Analysis: calculated for C_22_H_26_N_2_O_4_: C 69.09, H 6.85, N 7.32, O 16.73%; found: C 69.21, H.7.01, N 23.78%.

### Refinement

H atoms bound to C atoms were placed in calculated positions and treated as riding on their parent atoms, with C—H = 0.93 Å (aromatic C), C—H = 0.97Å (methylene C), and with *U*_iso_(H) = 1.2Ueq(C) or C—H = 0.96 Å (methly C) and with *U*_iso_(H) = 1.5Ueq(C). The H atoms attached to the O atoms were found from the Fourier difference map and the O–H bonds are refined in the normal range with *U*_iso_(H) = 1.5Ueq(O).

### Crystal data

**Table:**

C_22_H_26_N_2_O_4_	*F*(000) = 816
*M**_r_* = 382.45	*D*_x_ = 1.219 Mg m^−^^3^
Monoclinic, *P*2_1_/*c*	Mo *K*α radiation, λ = 0.71073 Å
Hall symbol: -P 2ybc	Cell parameters from 4748 reflections
*a* = 15.014 (3) Å	θ = 3.1–27.5°
*b* = 12.029 (2) Å	µ = 0.08 mm^−^^1^
*c* = 12.099 (2) Å	*T* = 293 K
β = 107.54 (3)°	Block, yellow
*V* = 2083.5 (6) Å^3^	0.22 × 0.20 × 0.15 mm
*Z* = 4	

### Data collection

**Table:**

Rigaku R-AXIS RAPID diffractometer	2352 reflections with *I* > 2σ(*I*)
Radiation source: fine-focus sealed tube	*R*_int_ = 0.062
graphite	θ_max_ = 27.5°, θ_min_ = 3.1°
Detector resolution: 10.000 pixels mm^-1^	*h* = −19→19
ω scans	*k* = −15→15
19987 measured reflections	*l* = −15→14
4748 independent reflections	

### Refinement

**Table:**

Refinement on *F*^2^	Primary atom site location: structure-invariant direct methods
Least-squares matrix: full	Secondary atom site location: difference Fourier map
*R*[*F*^2^ > 2σ(*F*^2^)] = 0.072	Hydrogen site location: inferred from neighbouring sites
*wR*(*F*^2^) = 0.164	H atoms treated by a mixture of independent and constrained refinement
*S* = 1.03	*w* = 1/[σ^2^(*F*_o_^2^) + (0.0509*P*)^2^ + 0.6508*P*] where *P* = (*F*_o_^2^ + 2*F*_c_^2^)/3
4748 reflections	(Δ/σ)_max_ = 0.001
259 parameters	Δρ_max_ = 0.13 e Å^−^^3^
1 restraint	Δρ_min_ = −0.18 e Å^−^^3^

### Special details

**Table:**

Geometry. All e.s.d.'s (except the e.s.d. in the dihedral angle between two l.s. planes) are estimated using the full covariance matrix. The cell e.s.d.'s are taken into account individually in the estimation of e.s.d.'s in distances, angles and torsion angles; correlations between e.s.d.'s in cell parameters are only used when they are defined by crystal symmetry. An approximate (isotropic) treatment of cell e.s.d.'s is used for estimating e.s.d.'s involving l.s. planes.
Refinement. Refinement of *F*^2^ against ALL reflections. The weighted *R*-factor *wR* and goodness of fit *S* are based on *F*^2^, conventional *R*-factors *R* are based on *F*, with *F* set to zero for negative *F*^2^. The threshold expression of *F*^2^ > σ(*F*^2^) is used only for calculating *R*-factors(gt) *etc*. and is not relevant to the choice of reflections for refinement. *R*-factors based on *F*^2^ are statistically about twice as large as those based on *F*, and *R*- factors based on ALL data will be even larger.

### Fractional atomic coordinates and isotropic or equivalent isotropic displacement parameters (Å^2^)

**Table:**

	*x*	*y*	*z*	*U*_iso_*/*U*_eq_	
C1	0.0604 (3)	0.6458 (3)	0.0711 (3)	0.1152 (12)	
H1A	0.0594	0.6532	−0.0083	0.173*	
H1B	0.1008	0.7015	0.1171	0.173*	
H1C	−0.0016	0.6553	0.0765	0.173*	
C2	0.0895 (3)	0.5459 (3)	0.4138 (4)	0.1002 (11)	
H2A	0.0743	0.5934	0.4661	0.120*	
C3	0.6339 (2)	0.4074 (3)	0.4551 (3)	0.0913 (10)	
H3A	0.6771	0.4377	0.4223	0.110*	
C4	0.2019 (2)	−0.1264 (2)	0.3541 (3)	0.0877 (9)	
H4A	0.2139	−0.1370	0.4368	0.105*	
H4B	0.1573	−0.1825	0.3140	0.105*	
C5	0.1206 (2)	0.4412 (3)	0.4475 (3)	0.0897 (9)	
H5A	0.1276	0.4182	0.5231	0.108*	
C6	0.2916 (2)	−0.1400 (2)	0.3237 (3)	0.0832 (9)	
H6A	0.2788	−0.1342	0.2404	0.100*	
H6B	0.3175	−0.2132	0.3475	0.100*	
C7	0.6257 (2)	0.5092 (3)	0.7826 (3)	0.1011 (11)	
H7A	0.6137	0.5225	0.8550	0.152*	
H7B	0.6185	0.5774	0.7395	0.152*	
H7C	0.6883	0.4821	0.7971	0.152*	
C8	0.5739 (2)	0.3277 (3)	0.3967 (2)	0.0801 (9)	
H8A	0.5761	0.3042	0.3243	0.096*	
C9	0.0802 (2)	0.5821 (2)	0.3029 (3)	0.0869 (9)	
H9A	0.0599	0.6542	0.2813	0.104*	
C10	0.36190 (19)	−0.0523 (2)	0.3828 (2)	0.0712 (8)	
H10A	0.4176	−0.0605	0.3587	0.085*	
H10B	0.3796	−0.0634	0.4660	0.085*	
C11	0.16120 (19)	−0.0121 (2)	0.3201 (3)	0.0805 (9)	
H11A	0.1046	−0.0040	0.3421	0.097*	
H11B	0.1449	−0.0038	0.2366	0.097*	
C12	0.10074 (19)	0.5126 (2)	0.2240 (3)	0.0721 (8)	
C13	0.14201 (18)	0.3684 (2)	0.3698 (2)	0.0645 (7)	
C14	0.6317 (2)	0.4439 (2)	0.5620 (2)	0.0732 (8)	
H14A	0.6731	0.4987	0.6009	0.088*	
C15	0.17636 (18)	0.2574 (2)	0.4061 (2)	0.0658 (7)	
H15A	0.1869	0.2370	0.4831	0.079*	
C16	0.23034 (17)	0.0781 (2)	0.3785 (2)	0.0605 (7)	
H16A	0.2407	0.0746	0.4624	0.073*	
C17	0.13111 (18)	0.4042 (2)	0.2567 (3)	0.0677 (7)	
C18	0.56880 (19)	0.3998 (2)	0.6112 (2)	0.0605 (7)	
C19	0.50689 (17)	0.3178 (2)	0.5532 (2)	0.0546 (6)	
C20	0.32359 (16)	0.0644 (2)	0.3545 (2)	0.0565 (6)	
H20A	0.3149	0.0794	0.2723	0.068*	
C21	0.50889 (17)	0.2810 (2)	0.44483 (19)	0.0558 (6)	
C22	0.44426 (17)	0.1969 (2)	0.3816 (2)	0.0574 (6)	
H22A	0.4441	0.1787	0.3068	0.069*	
N1	0.19248 (14)	0.18712 (18)	0.33625 (18)	0.0628 (6)	
N2	0.38790 (14)	0.14722 (17)	0.42452 (16)	0.0568 (5)	
O1	0.56134 (14)	0.42882 (16)	0.71764 (15)	0.0826 (6)	
O2	0.09388 (17)	0.53935 (17)	0.1121 (2)	0.1010 (7)	
O3	0.15024 (16)	0.33730 (17)	0.17764 (17)	0.0920 (7)	
H3	0.166 (3)	0.2728 (19)	0.211 (3)	0.138*	
O4	0.44555 (14)	0.27515 (16)	0.60379 (15)	0.0754 (6)	
H4	0.414 (2)	0.225 (3)	0.557 (3)	0.113*	

### Atomic displacement parameters (Å^2^)

**Table:**

	*U*^11^	*U*^22^	*U*^33^	*U*^12^	*U*^13^	*U*^23^
C1	0.121 (3)	0.082 (2)	0.148 (3)	0.027 (2)	0.048 (3)	0.034 (2)
C2	0.113 (3)	0.078 (2)	0.125 (3)	−0.003 (2)	0.058 (2)	−0.029 (2)
C3	0.093 (2)	0.111 (3)	0.081 (2)	−0.041 (2)	0.0441 (18)	−0.012 (2)
C4	0.082 (2)	0.0693 (19)	0.112 (2)	−0.0142 (16)	0.0286 (19)	0.0015 (18)
C5	0.105 (3)	0.081 (2)	0.095 (2)	−0.0020 (19)	0.049 (2)	−0.0140 (19)
C6	0.083 (2)	0.0656 (18)	0.098 (2)	0.0009 (16)	0.0235 (18)	−0.0063 (17)
C7	0.116 (3)	0.106 (2)	0.073 (2)	−0.028 (2)	0.0178 (19)	−0.0256 (19)
C8	0.086 (2)	0.099 (2)	0.0646 (17)	−0.0261 (18)	0.0367 (16)	−0.0097 (16)
C9	0.075 (2)	0.0583 (18)	0.130 (3)	−0.0048 (15)	0.036 (2)	−0.011 (2)
C10	0.0629 (18)	0.0709 (18)	0.0779 (18)	0.0088 (15)	0.0182 (14)	0.0011 (15)
C11	0.0510 (17)	0.082 (2)	0.105 (2)	−0.0047 (15)	0.0185 (16)	0.0027 (18)
C12	0.0568 (18)	0.0676 (19)	0.089 (2)	0.0003 (14)	0.0181 (15)	−0.0017 (17)
C13	0.0553 (16)	0.0637 (17)	0.0768 (18)	−0.0028 (13)	0.0231 (14)	−0.0080 (15)
C14	0.071 (2)	0.0743 (18)	0.0709 (18)	−0.0194 (15)	0.0154 (15)	−0.0041 (15)
C15	0.0591 (17)	0.0781 (19)	0.0619 (15)	−0.0067 (14)	0.0207 (13)	−0.0010 (15)
C16	0.0526 (16)	0.0638 (16)	0.0643 (15)	0.0046 (13)	0.0163 (12)	0.0075 (13)
C17	0.0513 (16)	0.0673 (18)	0.0813 (19)	0.0062 (14)	0.0150 (14)	−0.0084 (16)
C18	0.0653 (17)	0.0625 (16)	0.0514 (14)	0.0017 (14)	0.0142 (13)	0.0037 (13)
C19	0.0539 (15)	0.0601 (15)	0.0508 (14)	0.0007 (13)	0.0175 (12)	0.0066 (12)
C20	0.0523 (15)	0.0650 (16)	0.0519 (13)	−0.0002 (13)	0.0151 (12)	0.0026 (13)
C21	0.0546 (16)	0.0651 (16)	0.0475 (13)	−0.0021 (13)	0.0151 (12)	0.0028 (12)
C22	0.0566 (16)	0.0722 (17)	0.0446 (13)	0.0030 (13)	0.0170 (12)	0.0015 (13)
N1	0.0553 (13)	0.0680 (14)	0.0620 (13)	0.0058 (11)	0.0129 (11)	−0.0021 (12)
N2	0.0513 (13)	0.0666 (13)	0.0527 (11)	−0.0026 (10)	0.0157 (10)	0.0019 (10)
O1	0.0954 (15)	0.0916 (14)	0.0620 (11)	−0.0214 (12)	0.0258 (10)	−0.0159 (11)
O2	0.1142 (18)	0.0841 (15)	0.1000 (17)	0.0303 (13)	0.0250 (14)	0.0171 (13)
O3	0.1182 (18)	0.0836 (14)	0.0708 (13)	0.0356 (13)	0.0233 (12)	0.0012 (11)
O4	0.0820 (14)	0.0921 (15)	0.0601 (11)	−0.0245 (11)	0.0335 (10)	−0.0087 (10)

### Geometric parameters (Å, °)

**Table:**

C1—O2	1.410 (3)	C10—H10A	0.9700
C1—H1A	0.9600	C10—H10B	0.9700
C1—H1B	0.9600	C11—C16	1.521 (3)
C1—H1C	0.9600	C11—H11A	0.9700
C2—C5	1.363 (4)	C11—H11B	0.9700
C2—C9	1.378 (4)	C12—O2	1.365 (3)
C2—H2A	0.9300	C12—C17	1.399 (4)
C3—C8	1.358 (4)	C13—C17	1.396 (4)
C3—C14	1.377 (4)	C13—C15	1.452 (4)
C3—H3A	0.9300	C14—C18	1.367 (3)
C4—C6	1.509 (4)	C14—H14A	0.9300
C4—C11	1.510 (4)	C15—N1	1.269 (3)
C4—H4A	0.9700	C15—H15A	0.9300
C4—H4B	0.9700	C16—N1	1.459 (3)
C5—C13	1.392 (4)	C16—C20	1.522 (3)
C5—H5A	0.9300	C16—H16A	0.9800
C6—C10	1.511 (4)	C17—O3	1.346 (3)
C6—H6A	0.9700	C18—O1	1.371 (3)
C6—H6B	0.9700	C18—C19	1.392 (3)
C7—O1	1.425 (3)	C19—O4	1.351 (3)
C7—H7A	0.9600	C19—C21	1.393 (3)
C7—H7B	0.9600	C20—N2	1.466 (3)
C7—H7C	0.9600	C20—H20A	0.9800
C8—C21	1.397 (3)	C21—C22	1.450 (3)
C8—H8A	0.9300	C22—N2	1.268 (3)
C9—C12	1.371 (4)	C22—H22A	0.9300
C9—H9A	0.9300	O3—H3	0.876 (18)
C10—C20	1.516 (3)	O4—H4	0.86 (3)
			
O2—C1—H1A	109.5	C4—C11—H11B	109.4
O2—C1—H1B	109.5	C16—C11—H11B	109.4
H1A—C1—H1B	109.5	H11A—C11—H11B	108.0
O2—C1—H1C	109.5	O2—C12—C9	125.5 (3)
H1A—C1—H1C	109.5	O2—C12—C17	114.9 (3)
H1B—C1—H1C	109.5	C9—C12—C17	119.6 (3)
C5—C2—C9	120.6 (3)	C5—C13—C17	119.1 (3)
C5—C2—H2A	119.7	C5—C13—C15	120.5 (3)
C9—C2—H2A	119.7	C17—C13—C15	120.4 (2)
C8—C3—C14	121.0 (3)	C18—C14—C3	120.0 (3)
C8—C3—H3A	119.5	C18—C14—H14A	120.0
C14—C3—H3A	119.5	C3—C14—H14A	120.0
C6—C4—C11	110.5 (2)	N1—C15—C13	122.2 (3)
C6—C4—H4A	109.6	N1—C15—H15A	118.9
C11—C4—H4A	109.6	C13—C15—H15A	118.9
C6—C4—H4B	109.6	N1—C16—C11	109.8 (2)
C11—C4—H4B	109.6	N1—C16—C20	108.3 (2)
H4A—C4—H4B	108.1	C11—C16—C20	111.9 (2)
C2—C5—C13	120.5 (3)	N1—C16—H16A	108.9
C2—C5—H5A	119.8	C11—C16—H16A	108.9
C13—C5—H5A	119.8	C20—C16—H16A	108.9
C4—C6—C10	110.9 (2)	O3—C17—C13	121.7 (2)
C4—C6—H6A	109.5	O3—C17—C12	118.5 (3)
C10—C6—H6A	109.5	C13—C17—C12	119.7 (3)
C4—C6—H6B	109.5	C14—C18—O1	125.0 (2)
C10—C6—H6B	109.5	C14—C18—C19	120.0 (2)
H6A—C6—H6B	108.0	O1—C18—C19	115.0 (2)
O1—C7—H7A	109.5	O4—C19—C18	118.9 (2)
O1—C7—H7B	109.5	O4—C19—C21	121.1 (2)
H7A—C7—H7B	109.5	C18—C19—C21	120.0 (2)
O1—C7—H7C	109.5	N2—C20—C10	111.1 (2)
H7A—C7—H7C	109.5	N2—C20—C16	107.46 (19)
H7B—C7—H7C	109.5	C10—C20—C16	111.4 (2)
C3—C8—C21	120.3 (3)	N2—C20—H20A	108.9
C3—C8—H8A	119.9	C10—C20—H20A	108.9
C21—C8—H8A	119.9	C16—C20—H20A	108.9
C12—C9—C2	120.5 (3)	C19—C21—C8	118.7 (2)
C12—C9—H9A	119.8	C19—C21—C22	121.2 (2)
C2—C9—H9A	119.8	C8—C21—C22	120.1 (2)
C6—C10—C20	112.1 (2)	N2—C22—C21	122.4 (2)
C6—C10—H10A	109.2	N2—C22—H22A	118.8
C20—C10—H10A	109.2	C21—C22—H22A	118.8
C6—C10—H10B	109.2	C15—N1—C16	119.8 (2)
C20—C10—H10B	109.2	C22—N2—C20	119.2 (2)
H10A—C10—H10B	107.9	C18—O1—C7	117.2 (2)
C4—C11—C16	111.1 (2)	C12—O2—C1	118.4 (3)
C4—C11—H11A	109.4	C17—O3—H3	107 (2)
C16—C11—H11A	109.4	C19—O4—H4	106 (2)

### Hydrogen-bond geometry (Å, °)

**Table:**

*D*—H···*A*	*D*—H	H···*A*	*D*···*A*	*D*—H···*A*
O3—H3···N1	0.88 (2)	1.77 (2)	2.572 (3)	150 (3)
O4—H4···N2	0.86 (3)	1.80 (3)	2.587 (3)	152 (3)

## References

[bb1] Aslantaş, M., Tümer, M., Şahin, E. & Tümer, F. (2007). *Acta Cryst.* E**63**, o644–o645.

[bb5] Rigaku (1998). *RAPID-AUTO* Rigaku Corporation, Tokyo, Japan.

[bb2] Rigaku/MSC (2002). *CrystalClear* Rigaku/MSC Inc., The Woodlands, Texas, USA.

[bb3] Sheldrick, G. M. (2008). *Acta Cryst.* A**64**, 112–122. 10.1107/S0108767307043930 18156677

[bb4] Tozzo, E., Romera, S., Santos, M. P., Muraro, M., Santos, R. H. De A., Liao, L. M., Vizotto, L. & Dockal, E. R. (2008). *J. Mol. Struct.* **876**, 110–120.

